# Chlorin e6声动力对人肺腺癌细胞SPCA-1生长的作用

**DOI:** 10.3779/j.issn.1009-3419.2010.03.03

**Published:** 2010-03-20

**Authors:** 锐年 郑, 为民 张, 晓怀 王, 慧洁 高

**Affiliations:** 1 510010 广州，广州军区广州总医院肿瘤科 Department of Medical Oncology, Guangzhou General Hospital of Guangzhou Military Command, Guangzhou 510010, China; 2 510515 广州，南方医科大学研究生院 Graduate School, Nanfang Medical University, Guangzhou 510515, China

**Keywords:** 二氢卟吩e6, 肺肿瘤, 单核细胞, 细胞增殖, Chlorin e6, Lung neoplasms, Monocytes, Cell proliferation

## Abstract

**背景与目的:**

声动力治疗（sonodynamic therapy, SDT）是通过超声波激活肿瘤细胞内聚集的声敏剂来治疗肿瘤的一种新方法。本实验用二氢卟吩e6（Chlorin e6）为声敏剂，通过超声波激活，研究其对人肺腺癌细胞SPCA-1生长的作用。

**方法:**

超声与Chlorin e6单独及联合处理SPCA-1细胞和正常人外周血单核细胞（normal peripheral mononuclear cell, PMNC），6 h后四甲基偶氮唑盐（3-[4, 5-dimethylthiazol-2-yl]-2, 5-diphenyl -tetrazolium bromide, MTT）显色分光光度法测细胞增殖，倒置显微镜观察细胞形态。

**结果:**

1.0 MHz频率超声强度1.0 W/cm^2^-2.0 W/cm^2^作用60 s呈强度依赖性抑制SPCA-1和PMNC细胞生长。Chlorin e6（0.4 mg/mL-3.2 mg/mL）浓度依赖性地抑制SPCA-1和PMNC细胞生长。与单纯超声（1.0 W/cm^2^×60 s×1.0 MHz）和Chlorin e6（0.05 mg/mL-0.2 mg/mL）相比，超声联合Chlorin e6对SPCA-1细胞生长抑制作用明显增强（*P* < 0.05），对PMNC细胞抑制作用无明显变化（*P* > 0.05）。细胞形态显示，与单纯超声（1.0 W/cm^2^×60 s×1.0 MHz）和Chlorin e6（0.2 mg/mL）相比，超声联合Chlorin e6组SPCA-1细胞死亡率明显增多（*P* < 0.05）。

**结论:**

超声联合Chlorin e6声动力可特异性抑制人肺腺癌SPCA-1细胞生长，Chlorin e6有望成为一个用于声动力治疗非小细胞肺癌的新型声敏剂。

## Abstract (en)

**Background and objective:**

Sonodynamic therapy (SDT) is a promising new approach for cancer therapy. The purpose of this study is to detect the effects of SDT on the cell proliferation of human lung adenocarcinoma cell SPCA-1, using Chlorin e6 as a sonosensitizing agent activated by ultrasound.

**Methods:**

SPCA-1 and normal peripheral mono- nuclear cell (PMNC) were treated with ultrasound or Chlorin e6 alone and combined. Cell proliferation was determined by MTT assay, and cell morphology was studied by inverted microscope after 6 h treated.

**Results:**

1.0 MHz ultrasound (1.0 W/ cm^2^-2.0 W/cm^2^×60 s) and Chlorin e6 (0.4 mg/mL-3.2 mg/mL) inhibited the cell proliferation of both SPCA-1 and PMNC cells in a intensity- and a dose-dependent manner respectively. Compared with the ultrasound (1.0 W/cm^2^×60 s) or Chlorin e6 (0.05 mg/mL-0.2 mg/mL) alone, the inhibitory effect on the cell proliferation was remarkably increased by the combination of ultrasound and chlorin e6 in SPCA-1 cells (P < 0.05), but no same effect was observed in PMNC cells (P > 0.05). Compared with the ultrasound (1.0 W/cm^2^×60 s) or chlorin e6 (0.2 mg/mL) alone, the combination treatment of ultrasound with Chlo- rin e6 induced more necrotic cells in SPCA-1 cells (P < 0.05).

**Conclusion:**

There was a significant selectively inhibitory effect of sonodynamic effect with Chlorin e6 on the SPCA-1 cell growth. Chlorin e6 may be a promising sonosensitizing agent for the treatment of non-small cell lung cancer.

肺癌是肿瘤中威胁人类生命健康的头号杀手，70%患者在诊断时已是局部晚期或有远处转移。第三代化疗药物与铂类的联合化疗是晚期非小细胞肺癌（non-small cell lung cancer, NSCLC）的标准治疗，存在的问题是疗效到达平台：有效率为25%-35%，中位生存期为8个月-10个月。以表皮生长因子受体（epidermal growth factor receptor, EGFR）及其细胞信号转导为靶点的肺癌靶向治疗虽不良反应小，但其有效性与*EGFR*基因突变有关，总体人群有效率低，且中位耐药时间为8个月-10个月。化疗与抗血管生成等靶向药物联合治疗亦仅延长生存1个月-3个月。因此，寻找高效、低毒的治疗肺癌新方法是医学界面临的重要问题。

声动力疗法（sonodynamic, SDT）是在光动力疗法（photodynamic therapy, PDT）基础上发展起来的一种肿瘤治疗新理念、新方法。其机制是口服或静脉注射声敏剂，声敏剂通过血流特异性在肿瘤细胞内聚集，然后用一定频率和强度的超声波照射肿瘤位置，通过声能激活声敏剂，使其产生一系列化学反应（如产生单态氧），导致肿瘤细胞的不可逆性损伤^[1, 2]^。与光动力疗法（激光激活光敏剂，产生单态氧或者自由基，从而起到抗肿瘤作用）相比，超声对人体组织有很强的无创穿透能力，无需借助其他介入手段即可对深部肿瘤进行治疗，故有着广阔的发展前景。虽大量的临床前基础研究显示SDT可高效特异杀伤肿瘤^[3-5]^，目前SDT尚未被批准用于临床的主要原因之一是既往采用的声敏剂是血卟啉类的光敏剂，该类光（声）敏剂不但肿瘤特异性差，而且在正常组织内排泄慢，注射后需避光近1个月。因此，近年来SDT的研究多集中于发掘新型声敏剂。遗憾的是，大多数声敏剂仍未能解决两大问题：肿瘤的特异性及非肿瘤组织清除率，故仍未适用于人体。

二氢卟吩（Chlorin e6）是一种结构类似于血卟啉的叶绿素降解产物。1974年Yamamoto^[6]^首次采用激光辐照Chlorin e6光动力治疗艾氏细胞瘤，杀伤作用明显。之后其光动力抗肿瘤效应经大量的动物实验得到证实，且具低毒、肿瘤特异性聚集高、正常组织清除快、光毒副作用低的优点^[7-9]^，是一个具有诱人前景的光敏剂，Chlorin e6已完成Ⅰ期临床试验光动力治疗黑色素瘤皮肤转移瘤^[10]^。鉴于多数光敏剂亦同时具声动力作用，本研究利用频率1.0 MHz的超声激活Chlorin e6，观察Chlorin e6声动力对人肺腺癌SPCA-1细胞生长的抑制作用，旨在探索Chlorin e6作为声敏剂治疗NSCLC的可能。

## 1 材料与方法

### 1.1 主要材料

#### 1.1.1 细胞系

人肺腺癌细胞SPCA-1细胞株由中科院上海细胞生物学研究所提供。正常人外周血单核细胞（normal peripheral mononuclear cell, PMNC）细胞株通过COBE spectra血细胞分离机收集于35岁健康成人自愿者。

#### 1.1.2 主要试剂和仪器

四甲基偶氮唑盐（3-[4, 5-dimeth-ylthiazol-2-yl]-2, 5-diphenyl-tetrazolium bromide, MTT）及二甲基亚砜（dimethyl sulfoxide, DMSO）购于Sigma公司。RPMI-1640培养液、小牛血清均为Gibco产品。Chlorin e6由EEC Bio-Tech Co. Ltd（广州）公司提供。其分子结构为：Sodium13-carboxy-17-[2-carboxyethyl]-15-carboxymethyl-17, 18-trans-dihydro-3-vinyl-8-ethyl-2, 7, 12, 18-tetramethyl-porphyrin（图 1A）。Chlorin e6是一种光敏剂，经我们检测Chlorin e6在400 nm和653 nm（可见红光区）处有两个吸收峰（图 1B），与国外报道的一致^[11]^。酶标仪为澳大利亚Biocell lt-2产品。倒置显微镜为日本Olympus产品。超声治疗仪由北京天使公司提供（频率为1.0 MHz，声强为0.1 W/cm^2^-2.2 W/cm^2^）。COBE spectra血细胞分离机为美国GAMBRO. BCT公司产品。

**1:**
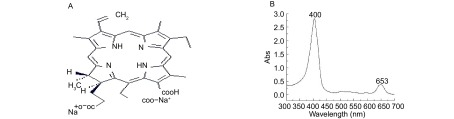
Chlorin e6的结构图以及紫外-可见光吸收光谱 The structure diagram and absorbance spectra of chlorin e6

### 1.2 方法

#### 1.2.1 细胞培养

人肺腺癌细胞SPCA-1置于含10%小牛血清、100 U/mL青霉素和100 U/mL链霉素的RPMI 1640培养液中，5%CO_2_培养箱37 ℃培养，每2-3天传代一次，每次实验前取状态良好的对数生长期细胞，经胰蛋白酶（Invitrogen公司）/乙二胺四乙酸（ethylene diamine tetraacetic acid, EDTA）消化液消化后备用。PMNC细胞由血细胞分离机收集后将其置于含10%小牛血清、100 U/mL青霉素和100 U/mL链霉素的RPMI-1640培养液中，5%CO_2_培养箱37 ℃培养，24 h内备用。

#### 1.2.2 MTT法检测细胞活性

取0.5 mL对数生长期SPCA-1细胞（2.5×10^5^/mL）和24 h内备用的PMNC（1.0×10^6^/ mL），移入2 mL冻存管，按不同的实验要求加入不同浓度Chlorin e6，孵育45 min，将冻存管浸入盛有蒸馏水的容器中，超声平面探头置于冻存管底部外相距2 mm处，超声辐照60 s后（图 2），将细胞悬液按每孔100 μL移入96孔板（每组3个平行复孔），之后继续培养6 h，每孔加入MTT（5 g/L），培养4 h后弃上清，每孔加入DMSO 100 μL，振荡10 min，用自动酶标仪检测在570 nm波长下各孔光密度值（optical density, OD），细胞存活率（%）=（OD实验组/OD对照组）×100%。实验重复6次。

**2:**
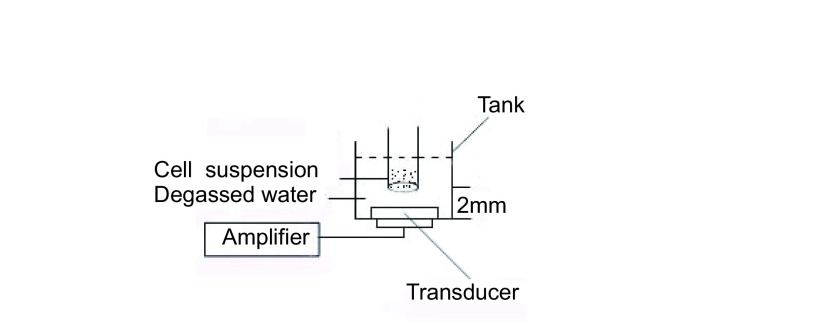
超声辐照实验装置图 Ultrasonic exposure system

#### 1.2.3 细胞形态变化的观察

倒置显微镜观察细胞存活情况：每孔随机选择1个高倍视野（×400），计算每个高倍镜视野死亡细胞的百分数。细胞死亡率（%）=（每个高倍视野死细胞数/每个高倍视野总细胞数）×100%。

#### 1.2.4 实验分组

超声频率固定为1.0 MHz，超声辐照时间固定为60 s。各组处理后6 h测细胞活性及观察细胞形态。超声组：声强设定为0 W/cm^2^（对照组）、0.5 W/ cm^2^、1.0 W/cm^2^、1.5 W/cm^2^、2.0 W/cm^2^；Chlorin e6组：Chlorin e6浓度设定为0mg/mL（对照组）、0.1 mg/mL、0.2 mg/mL、0.4 mg/mL、0.8 mg/mL、1.6 mg/mL、3.2 mg/ mL；声动组：声强设为1.0 W/cm^2^，分为对照组（C）、超声组（U1.0）、Chlorin e6 0.2 mg/mL组（E0.2）、超声+Chlorin e6组（U1.0 E0.05、U1.0 E0.1、U1.0 E0.2）。

### 1.3 统计学处理

实验数据采用SPSS 13.0软件进统计学分析，实验结果以Mean±SD表示，相关各组间采用*One-way ANOVA*检验，*P* < 0.05判定为差异有统计学意义。

## 2 结果

### 2.1 超声及Chlorin e6对细胞生长的影响

1.0 MHz超声（1.0-2.0）W/cm^2^×60 s呈强度依赖性抑制SPCA-1和PMNC细胞生长（图 3A），超声抑制50%SPCA-1和PMNC细胞生长的强度分别为：1.45 W/cm^2^、1.44 W/cm^2^，无统计学差异（*P* > 0.05）。Chlorin e6（0.4-3.2）mg/mL呈浓度依赖性地抑制SPCA-1和PMNC细胞生长（图 3B）。Chlorin e6抑制SPCA-1和PMNC细胞生长的IC_50_值分别为：0.79 mg/ mL、0.97 mg/mL，无统计学差异（*P* > 0.05）。

**3:**
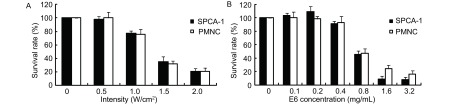
超声（A）及Chlorin e6（B）对SPCA-1及PMNC细胞生长的影响 Effects of ultrasound (A) and Chlorin e6 (B) on the proliferation of SPCA-1 and PMNC cells

### 2.2 超声与Chlorin e6联合作用对细胞生长的影响

1.0 W/cm^2^单纯超声作用时，SPCA-1和PMNC细胞存活率分别为79.6%、78.1%，与正常对照组相比，均有统计学差异（*P* < 0.05），SPCA-1和PMNC细胞间比较无统计学差异（*P* > 0.05）。图 4所示，0.05 mg/mL、0.1 mg/mL和0.2 mg/mL三个不同浓度Chlorin e6单纯作用对SPCA-1和PMNC细胞生长均无明显作用（*P* > 0.05）；1.0 W/cm^2^超声联合该三个不同浓度Chlorin e6作用时，SPCA-1细胞存活率分别为66.1%、53.0%和28.0%，PMNC细胞存活率分别为78.7%、80.5%和75.2 %。与单纯超声和Chlorin e6相比，超声联合Chlorin e6对SPCA-1细胞生长的抑制有统计学差异（*P* < 0.05），对PMNC细胞存活率无统计学差异（*P* > 0.05）。

**4:**
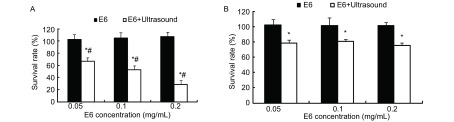
超声联合Chlorin e6声动力对SPCA-1（A）及PMNC（B）细胞生长的影响 The sonodynamic effects, using chlorin e6 as a sonosensitizing agent activated by ultrasound, on the proliferation of SPCA-1 and PMNC cells

### 2.3 倒置显微镜观察超声与Chlorin e6单独及联合作用对SPCA-1细胞存活的影响

高倍镜下，圆形、透亮、胞膜完整、胞浆丰富的细胞视为活细胞；多形、不透亮、胞膜破裂、胞浆流失的细胞视为死细胞（图 5）。C组、U1.0组、E0.2组、U1.0E0.2组SPCA-1细胞死亡率分别为1.6%、22.0%、2.3%、69.8%。与单纯超声的U1.0组和单纯Chlorin e6的E0.2组比，超声联合Chlorin e6的U1.0E0.2组SPCA-1细胞死亡率明显升高（*P* < 0.05）。

**5:**
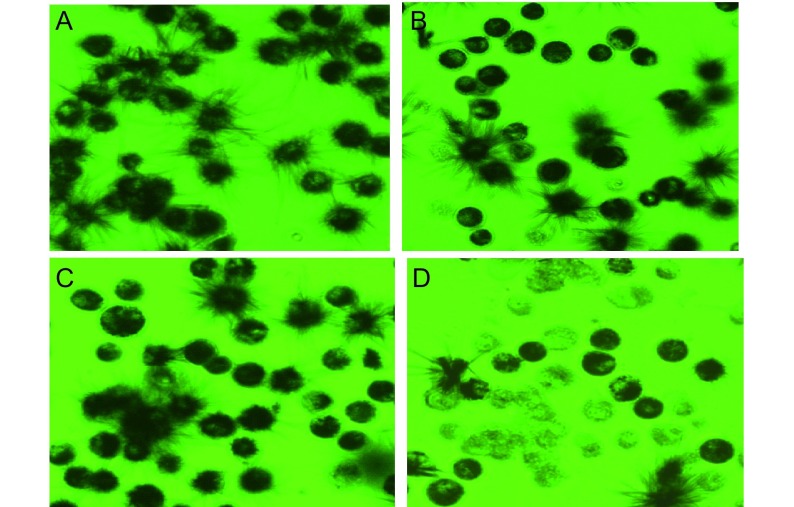
倒置显微镜观察超声、Chlorin e6单独及联合声动力治疗对SPCA-1细胞存活影响（×400） Morphological analysis of SPCA-1 cell survival induced by ultrasound and chlorin e6 alone or combination treatment (×400)

## 3 讨论

本研究首次将已证实具低毒、肿瘤特异性聚集高、正常组织清除快、光毒副作用低等优点的光敏剂Chlorin e6作为声敏剂对NSCLC细胞进行声动力研究，结果显示，1.0 MHz频率超声强度1.0 W/cm^2^-2.0 W/cm^2^作用60 s强度依赖性地抑制SPCA-1和PMNC细胞生长，说明一定频率和强度的超声不仅能损伤肿瘤细胞还能损伤正常细胞，与国内外报道相同^[12-14]^。其抑制50%SPCA-1和PMNC细胞生长的超声强度相似，说明超声对NSCLC细胞和正常单核细胞的损伤无明显差别，这与既往超声对其他肿瘤的研究不同。既往研究显示，肿瘤细胞相比正常细胞更易受超声损伤^[12]^：人肺癌、乳腺癌以及黑色素瘤细胞相比人包皮成纤维细胞以及人羊水膜上皮细胞更易受超声损伤^[13]^，成人T细胞白血病MT-2细胞相比正常人外周单核PMNC细胞更易受超声损伤^[14]^。可能的原因是：既往的研究是肿瘤细胞与同源的正常细胞相比，而本研究是上皮来源的人肺腺癌SPCA-1细胞与血液系统的正常单核细胞PMNC相比，血液系统的细胞较上皮细胞对理化作用更敏感，抵消了肿瘤细胞更易受超声损伤的作用；本研究所采用的超声频率与既往研究不同，不同频率的超声强度作用可能不同。其确切可能存在的原因有待进一步证实。

本实验Chlorin e6浓度依赖性地抑制SPCA-1细胞生长，Chlorin e6原本是光敏剂，既往研究显示：methylene blue、toluidine blue以及victoria blue等光敏剂均呈浓度依赖性地抑制肿瘤细胞生长^[15]^。本研究首次比较了Chlorin e6对肿瘤细胞和正常PMNC细胞的作用，其抑制SPCA-1及P M N C细胞生长的IC_50_值无明显差别，结果说明Chlorin e6本身对SPCA-1及PMNC细胞的杀伤作用相似，对SPCA-1细胞不具有靶向性。

理论上观察超声声动力作用最好选择对正常PMNC细胞不存在损伤的超声强度和Chlorin e6浓度。本实验选择0.05 mg/mL-0.2 mg/mL Chlorin e6做声动力研究是基于此浓度范围Chlorin e6对正常PMNC细胞不存在抑制效应，但本实验基于以下原由选择对正常PMNC约20%损伤的1.0 W/cm^2^超声强度进行声动力研究：1.0 W/cm^2^为临床常用超声强度^[16, 17]^；超声需达到一定强度产生空化效应时，才能激活声敏剂产生抗肿瘤效应，不同的声敏剂被激活所要求的超声强度不同^[18, 19]^。本研究的预实验显示，1.0 W/cm^2^超声强度才能激活Chlorin e6产生抗SPCA-1细胞生长作用。

本实验结果证实，超声可以激活Chlorin e6起特异性抗肿瘤作用。相比单纯超声和Chlorin e6，超声联合Chlorin e6组明显抑制SPCA-1细胞生长，对PMNC细胞生长的抑制效应无明显增强。本研究结果与Uchida等^[14]^用Photofrin为声敏剂报道的相似，他们发现超声联合Photofrin对成人T细胞白血病MT-2细胞生长的抑制效应明显增强，对PMNC细胞生长抑制效应无明显变化。说明超声联合Chlorin e6声动力对SPCA-1细胞生长的作用具靶向性。形态学上表现为SPCA-1细胞死亡率明显增强，说明Chlorin e6声动力抑制SPCA-1细胞生长作用是通过直接杀伤肿瘤细胞。

目前SDT的研究主要是声敏剂的开发，一方面，在血卟啉的基础上进行各种结构调整或化学修饰，产生如ATX-10、ATX-70、DCPH-P-Na I^[20]^等衍生物，降低了光毒性；另一方面，出现抗癌药物^[21]^、抗炎药物^[22]^等非卟啉类声敏剂，在超声照射下对肿瘤有一定的杀伤效果。鉴于既往的研究已显示Chlorin e6具低毒、肿瘤特异性聚集高、正常组织清除快和光毒副作用低等优点^[7-9]^。本研究Chlorin e6具声动力作用的结果表明Chlorin e6有望成为一个用于声动力治疗NSCLC的新型声敏剂，其作用有待在整体动物加以证实，是否具有普遍性有待在其他肺癌细胞、其他肿瘤细胞加以证实，是否优于其他声敏剂有待与其他光声敏剂比较。
